# A comparative study of defibrillation and cardiopulmonary resuscitation performance during simulated cardiac arrest in nursing student teams

**DOI:** 10.1186/1757-7241-20-23

**Published:** 2012-04-02

**Authors:** Sissel I Eikeland Husebø, Conrad A Bjørshol, Hans Rystedt, Febe Friberg, Eldar Søreide

**Affiliations:** 1 Department of Health Studies, Faculty of Social Sciences, University of Stavanger, Stavanger 4036, Norway; 2 Department of Anaesthesiology and Intensive Care, Stavanger University Hospital, PO Box 8100, Stavanger 4068, Norway; 3 Department of Education, Communication and Learning, University of Gothenburg, Läroverksgatan 15, PO Box 300, Gothenburg SE 405 30, Sweden; 4 Institute of Health and Care Sciences, Sahlgrenska Academy at University of Gothenburg, PO Box 100, Gothenburg S-405 30, Sweden

**Keywords:** Defibrillation, Cardiopulmonary resuscitation, Patient simulation, Nursing students

## Abstract

**Background:**

Although nurses must be able to respond quickly and effectively to cardiac arrest, numerous studies have demonstrated poor performance. Simulation is a promising learning tool for resuscitation team training but there are few studies that examine simulation for training defibrillation and cardiopulmonary resuscitation (D-CPR) in teams from the nursing education perspective. The aim of this study was to investigate the extent to which nursing student teams follow the D-CPR-algorithm in a simulated cardiac arrest, and if observing a simulated cardiac arrest scenario and participating in the post simulation debriefing would improve team performance.

**Methods:**

We studied video-recorded simulations of D-CPR performance in 28 nursing student teams. Besides describing the overall performance of D-CPR, we compared D-CPR performance in two groups. Group A (n = 14) performed D-CPR in a simulated cardiac arrest scenario, while Group B (n = 14) performed D-CPR after first observing performance of Group A and participating in the debriefing. We developed a D-CPR checklist to assess team performance.

**Results:**

Overall there were large variations in how accurately the nursing student teams performed the specific parts of the D-CPR algorithm. While few teams performed opening the airways and examination of breathing correctly, all teams used a 30:2 compression: ventilation ratio.

We found no difference between Group A and Group B in D-CPR performance, either in regard to total points on the check list or to time variables.

**Conclusion:**

We found that none of the nursing student teams achieved top scores on the D-CPR-checklist. Observing the training of other teams did not increase subsequent performance. We think all this indicates that more time must be assigned for repetitive practice and reflection. Moreover, the most important aspects of D-CPR, such as early defibrillation and hands-off time in relation to shock, must be highlighted in team-training of nursing students.

## Introduction

Nurses and nursing students must be able to respond correctly in the event of a cardiac arrest both inside and outside hospitals [1-4]. Most nursing education institutions have resuscitation training within their curricula to meet these expectations and to ensure that students are competent at commencing life support in cases of cardiac arrest. In spite of this, previous studies in the nursing research literature have described poor retention of knowledge and skills in performing resuscitation [3,5-7]. Several educational methods of improving cardiopulmonary resuscitation (CPR) have been tried out but both content and methods lack standardization [3]. Nevertheless, simulation can be used to meet these demands by creating learning opportunities that are unavailable in clinical practice, such as defibrillation and CPR (D-CPR) [8,9].

Several studies have been performed using cardiac arrest simulation to improve resuscitation performance by nurses [7,10-13]. However, there are few studies from the nursing education perspective that examine simulation for learning CPR. A previous study demonstrated that the nursing students needed several simulations to perform CPR accurately [14]. Scherer et al. [15] examined pre-and post-test knowledge of cardiac arrest and found no difference between the experimental group (simulation) and control group (case study seminar). Two other studies measuring satisfaction and/or self-confidence of nursing students after a cardiac arrest simulation demonstrated that students rated the experience as positive, enjoyable and instructive [16] and perceived the design and implementation to be very satisfying [17]. A study in medical practice demonstrated that observing simulation and participating in the post simulation debriefing in combination with participating in simulation improved performance in resuscitation [18]. A study of the briefing part of simulation in nursing education concluded that presupposing a higher level of resuscitation skills than expected in nursing education might interfere with opportunities to learn from simulation experiences [19]. Husebø et al. [20] identified three phases in resuscitation teamwork corresponding to the three first steps in the BLS algorithm: stating unconsciousness, preparing for resuscitation and initiating resuscitation, but questions remain unanswered as to which extent the nursing student teams followed the D-CPR algorithm and if different conditions in simulation improved team performance.

In this study, our aim was to investigate the extent to which nursing student teams followed the D-CPR algorithm. Moreover, we wanted to examine if observing one simulated cardiac arrest scenario and participation in one debriefing could improve team performance of D-CPR in a subsequent simulation.

## Methods

### Study participants

We invited half the cohort (n = 81) of nursing students from a three-year nursing education program at the University of Stavanger, Norway, to participate in the study. The students were in their last semester. Five faculty members participated as facilitators. The study was approved by the Norwegian Social Science Data Services (NSD) and the University of Stavanger but the ethics committee of the Western part of Norway declined to consider the application because the study did not involve patients or relatives. All students received oral and written information about the study at the start of the semester, and all participants signed an informed consent before being included in the study, confidentiality having been guaranteed. All 81 nursing students asked (72 female and 9 male) agreed to participate in the study; the average age was 26 (range 22-49 years). Each of the 28 nursing student teams consisted of between two and four team members. Eight of the teams included both males and females, while the rest consisted of females only.

### Setting

The study took place in a simulation centre where the simulation environment resembled a room in an out-of-hospital rehabilitation unit. A full-size patient simulator (SimMan, Laerdal Medical Inc., Norway) was controlled by an operator in an adjacent room. The patient simulator was placed in a bed and exhibited clinical signs such as palpable pulses, breath movements and sounds. A speaker located in the mannequin's head transmitted the voice of the operator, thus giving the impression that the 'patient' could talk. The room was equipped with a training semi-automatic defibrillator (Heartstart FR2, Laerdal Medical Inc., Norway), oxygen, backboard, medications, an oro-pharyngeal airway and a bag-mask manual ventilator. The simulations were video-recorded by two separate video cameras.

### Design and research procedures

The results are based on video-recordings of 28 simulated cardiac arrest scenarios in nursing education. The data were collected in February and March 2008. We first conducted a descriptive study of the overall D-CPR performance in 28 nursing student teams. Further, we compared performance of D-CPR in two groups of nursing student teams. Group A (14 teams) performed D-CPR in a simulated cardiac arrest scenario, while Group B (14 teams) performed D-CPR after first observing performance of Group A and participating in the debriefing.

**Figure 1:**
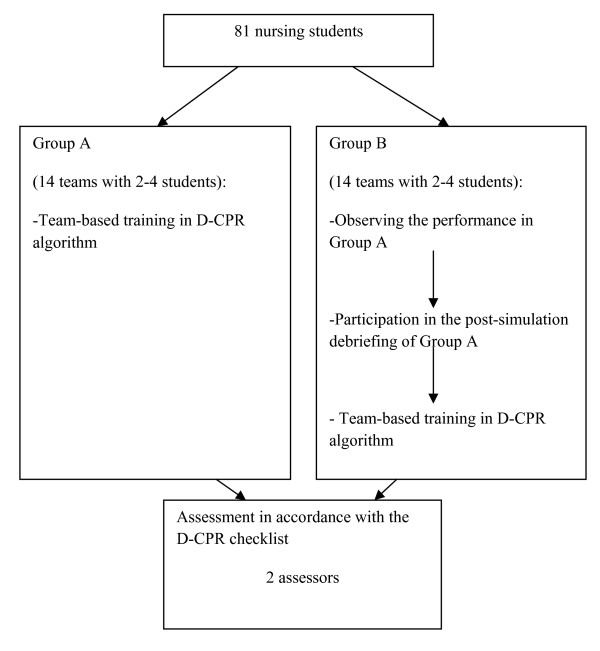
**Outline of research design**.

Three weeks prior to the simulation, all students received the team schedule list, a short description of the scenario and the learning objectives. The learning objectives were: 1) using the D-CPR guidelines in practice, and 2) optimizing teamwork in resuscitation teams. The D-CPR course was developed for the last semester in nursing education and comprised a two-hour lecture in class about the semi-automatic defibrillator. All teams were given 45 min of individual practical training in CPR and use of a semi-automatic defibrillator [21,22] before participating in the team-based simulation of a cardiac arrest. For each simulation, teams in Group A participated in the simulation scenario while teams in Group B were present in the room to observe. After completion of the post-simulation debriefing, Group B performed the simulation scenario, while Group A observed (Figure 1).

Prior to the simulation session, the facilitator gave all teams a 15-minute briefing regarding the function of the mannequin and the use of the medical equipment, and repeated the learning objectives. The following statement introduced the simulated scenario: The patient is a 71 year-old woman who has suffered an upper femur fracture and has been moved to an out-of-hospital rehabilitation unit without a physician present. The patient has a history of angina pectoris and is now complaining about chest pain. Your team are now required to manage this patient. The simulation started with the nurse entering the room to see if the patient had finished breakfast. After a few minutes with chest pain, the patient went into a cardiac arrest with a shock able rhythm. We discontinued the simulation 1 min after the first shock. Following the simulation, the students participated in the debriefing guided by the facilitator, who analysed team performance of D-CPR in relation to the learning objectives.

### Assessment of team performance and development of the D-CPR checklist

We developed a 23-item check list (D-CPR checklist) with a total score range from 0 to 19 (items 1-19) and the actual number of seconds was used for three items (item 20, 21 and 24) to assess D-CPR team performance because no D-CPR checklist in Norwegian existed (Additional file 1). The D-CPR checklist was based on the Cardiff test protocol [23] and the checklist developed by Kromann et al. [22].

The Cardiff Test for basic life support (BLS) and the use of an automated external defibrillator (AED) from 2000 has been developed from previous editions and uses criterion-referenced assessments to evaluate CPR and AED performance from analysis of video recordings and data drawn from a computer attached to a training mannequin. The European Resuscitation Guidelines for CPR and AED were revised in 2005, and the D-CPR checklist in this study was revised accordingly [21]. The checklist developed by Kromann et al. [22] was based on the Advanced Life Support Cardiac Arrest Scenario Test checklist from 2005 [24]. For this study it was adjusted to assessment of basic life support and use of AED.

The researchers collaboratively designed the D-CPR checklist to match the curriculum of the course and expected team performance. For items 1 to 19, "yes" was coded as 1 and "no" as 0 (19 possible points). For items 20-24, *time from discovery of unconsciousness until chest compressions started*, *time from discovery of unconsciousness until shock was delivered *and *hands-off time in relation to first shock *the actual number of seconds was used. Firstly, two researchers independently assessed the performance of Group A and B in four of the cardiac arrest simulations. This served to refine the checklist, adjust items and calibrate judgments. Secondly, the two researchers independently assessed team performance for each item of the checklist in the remaining 24 scenarios. Thereafter, three errors of time registration were corrected, as these were caused by miscalculations. Seven differences in relation to timing of unconsciousness (item 20-21) were kept unchanged as these were caused by the difference between the two researchers in defining the exact point of time for discovering unconsciousness. Item 24 was calculated as the sum of items 22 and 23, and a summary of points for all teams according to each of the 19 items in the D-CPR-checklist was made. Further, the points given to each team by the two assessors were summarized separately and the mean values of the two sums were calculated.

### Statistics

Paired samples *t*-test was used for analyses of variables with normal distribution, i.e. item 1 to 19, time from discovery of unconsciousness until chest compressions started (item 20) and time from discovery of unconsciousness until shock was delivered (item 21). The Wilcoxon signed rank test was used on item 24 (Hands-off time in relation to first shock) due to abnormal distribution. For parametric tests, the mean value with standard deviation (SD) was calculated. For non-parametric tests, the median values with interquartile range were calculated. All tests were two-sided and statistical significance was considered as *P *< 0.05. All analyses were performed with SPSS version 18 (Chicago, IL). Rater agreement, defined as the number of agreed assessments (x + y) divided by the number of agreed assessments + the number of disagreed assessments (z) [25], was calculated using the fraction: $\%=\frac{\text{x + y}}{\left(\text{x + y}\right)+\text{z}}$ (Additional file 2). However, this calculation has at least two weaknesses: it takes no account of where in the checklist the agreement was, and we would expect some agreement between the two raters by chance, even if they were guessing. We therefore calculated inter-rater reliability of the video assessment with kappa and linear weighting using VassarStats^a ^(Additional file 2). It has been proposed that a kappa score of 0.81-1.00 indicates very good agreement, 0.41-0.80 moderate to good agreement, 0.21-0.40 fair agreement and below 0.20 poor agreement [26].

### Reliability of the D-CPR checklist

Rater agreement for assessment of the D-CPR checklist was 0.88 $\left(88\%=\frac{318+149}{\left(318+149\right)+65}\right)$ indicating a reliable checklist. Items regarding application of skills and medical devices received highest agreement between the two assessors (e.g. item 1. *Checked response verbally*, item 3. *Opened the airways*, item 5. *Verbally stated cardiac arrest*, and item 9. *Counted aloud*), whereas items regarding subjective data of D-CPR (e.g. item 2. *Checked response by shaking*, item 4. *Checked breathing for a max. 10 s*., and item 18. *Said all away from the patient*) received lowest agreement (Additional file 2). The inter-rater reliability is shown in Additional file 2. There was very good or good strength agreement for nine items. However, there was fair strength of agreement on checking response by shaking, checking breathing and standing on their toes (Additional file 2). Differences between rater 1 and rater 2 in time variables (item 20, 21 and 24) demonstrated that one rater (rater 2) consistently assessed time intervals longer than the other (Additional file 3).

## Results

### Team performance of D-CPR

**Figure 2:**
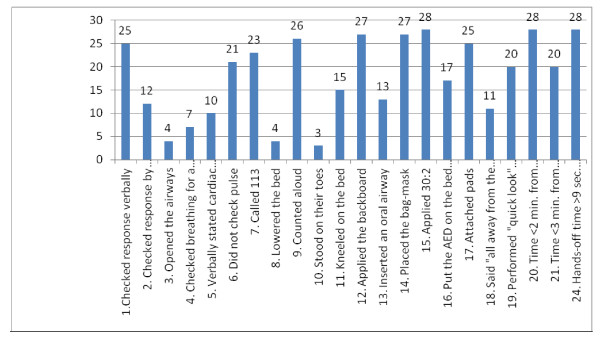
**The number of nursing student teams (n = 28) that followed the D-CPR checklist**. Teams for which there is disagreement in the assessed items are not included.

The nursing student teams achieved on average 59% of the D-CPR-checklist points $\left(x=\frac{\text{numbers}\;\text{of}\;\text{assessment}\;\text{with}\;\text{correct}\;\text{performance}\;\left(\text{n}=318\right)}{\text{number}\;\text{of}\;\text{teams}\;\left(n=28\right)\;\times \;\text{numbers}\;\text{of}\;\text{items}\;\left(\text{n}=19\right)}=59\%=\frac{318}{532}\right)$ (Additional file 2). Twenty-five (89%) of 28 teams performed *checking response verbally*, while only 12 (43%) teams *checked response by shaking *as prescribed by the guidelines (Figure 2). *Opened the airways *was the most poorly performed part of the D-CPR. Only seven teams (25%) checked breathing for a maximum 10 s. The most correctly performed part of the D-CPR was the use of a 30:2 ratio in compressions and ventilation (Figure 2). All but one (96%) team applied the backboard while they performed chest compressions. Twenty-five (89%) teams attached pads correctly. All nursing student teams started chest compressions < 2 min. from discovery of unconsciousness. Twenty (72%) teams took < 3 min. from discovery of unconsciousness until shock was delivered and none of the teams had < 9 s. hands-off time in relation to first shock (Figure 2).

### Differences between group A and B in D-CPR performance

When comparing Group A and Group B there were no significant differences in performance of D-CPR either in total points (item 1-19) or time variables (item 20, 21 and 24) (Table 1). There was a small increase in total points of D-CPR performance for Group B, but the changes were not significant. When it comes to *time from discovery of unconsciousness until chest compressions started *Group B took on average 2 s more than Group A (item 20). Group B also took on average 4 s more than Group A from *discovery of unconsciousness until shock was delivered *(item 21). *Hands-off time in relation to first shock *was more or less the same for both groups (item 24).

**Table 1:** D-CPR Checklist points and measured time intervals for Group A and Group B assessed via video-recordings

Items	Group A (n = 14)	Group B (n = 14)	P
D-CPR performance (item 1-19)*	12 (11-14)	13 (11-15)	.566

Time (sec.) from discovery of unconsciousness until chest compressions started (item 20)	36 ± 16	38 ± 16	.744

Time (sec.) from discovery of unconsciousness until shock was delivered (item 21)	145 ± 61	149 ± 44	.808

Hands-off time (sec.) in relation to first shock (item 24)*	33 (28-40)	33 (28-44)	.675

Mean values ± standard deviation (* median values with interquartile range)

## Discussion

The results of our study indicate that most of the nursing student teams did not perform the D-CPR-checklist accurately. We could also demonstrate that observing one simulated cardiac arrest scenario and participating in one post simulation debriefing did not show a significant improvement in performance of D-CPR. These findings can at first sight appear as somewhat surprising since feedback in the debriefing has been identified as the most important feature of simulation-based education and a necessary condition for changing performance [27,28]. In the debriefings, the active students were encouraged to reflect on and analyse their own performance leading to meaningful learning [29], whilst the observing nursing student teams were asked to reflect on the performance of the active teams and to keep that in mind for their subsequent simulation. Consequently, it might have been too demanding for these teams to both apply the D-CPR algorithm and simultaneously apply the newly acquired insight from their observations in the subsequent scenario [20]. A second explanation for the results of our study is that it takes more than one simulation and repetitive practice with feedback to perform D-CPR with accuracy [5,14,30,31]. In the present study the nursing student that were active in the second simulation had neither practiced themselves, nor got feedback on their performance before acting in the scenario.

In terms of D-CPR team performance, the findings are discouraging, in that procedures such as opening the airways and examination of breathing were not satisfactory. However, this does not mean that the nursing student teams failed to develop any of the D-CPR skills. The findings clearly demonstrated that the student teams achieved almost two-thirds (59%) of the D-CPR-checklist points by participating in a simulated cardiac arrest scenario. These findings are in line with the results of a study that compared a traditional, small-group D-CPR course with an Internet-based D-CPR course teaching basic life support [32]. Mäkinen et al. found that nurses receiving the traditional, small-group D-CPR course performed better than those receiving the Internet-based D-CPR course [32]. The importance of leadership in team performance has been demonstrated in previous research [33,34], but assessing leadership was beyond the scope and intent of this article.

The mean time from discovery of unconsciousness until chest compressions started was 36.3 s in Group A and 38 s in Group B. All teams took < 2 min. until chest compressions were initiated. Previous research has shown that the interval between discovery of unconsciousness until chest compressions start affects survival in cardiac arrest [35]. Holmberg et al. found that there was significantly increased survival at one month for patients who received CPR ≤ 2 min after collapse compared to patients who received CPR > 2 min. after collapse [35]. Our results indicate that all nursing student teams understood the importance of acting rapidly and starting chest compressions early to increase survival after cardiac arrest. If the teams had further trained in recognizing a cardiac arrest by stating unconsciousness and confirming abnormal breathing, the time to first chest compression could possibly have been further reduced.

Time from discovery of unconsciousness until shock is delivered influences survival in in-hospital cardiac arrest [36]. Herlitz et al. demonstrated that the overall survival rates were 72% for patients defibrillated within 3 min. after collapse on non-monitored wards [36]. In our study we found that, on average, all teams delivered first shock within the first three minutes, but the variance within the teams was large. This means that 8 teams took > 3 min. from discovery of unconsciousness until shock was delivered. These findings may be explained by confusion as to whether to deliver immediate defibrillation in case of witnessed cardiac arrest was appropriate or whether to execute 3 min. of CPR before defibrillation in case of non-witnessed cardiac arrest (Norwegian Resuscitation Council). This observation calls for a clear explanation of the correct algorithm to follow and more attention to early defibrillation in simulation-based D-CPR courses in nursing education; it should be specifically highlighted in the debriefing.

The results demonstrated that all teams spent too long hands-off time in relation to first shock. Hands-off time in relation to first shock is associated with decrease in survival [37]. A recent study aiming to define the optimal pre- and post-defibrillation compression pauses for out-of-hospital cardiac arrest revealed that hands-off time > 9 s. in relation to first shock decreased the return of spontaneous circulation [38]. The long time intervals concerning hands-off time in relation to first shock have educational implications.

In summary, this study has demonstrated that observing one simulation performed by another team and participating in its debriefing does not improve nursing student performance in a subsequent simulated cardiac arrest scenario. Further, this study has demonstrated that the five-hour program in D-CPR is insufficient for learning to perform D-CPR correctly. There is reason to believe that the program should include repetitive practice, feedback and testing of D-CPR performance, as previously demonstrated by Oermann et al. and Sutton et al. [30,31].

The major limitations of this study were its small sample size as well as its limitation of having been carried out in one nursing education institution, in one geographical location in Norway. Ideally, the design of the educational study should include a pre-test. However, in this study, a pre-test to examine if different conditions would change the team performance of D-CPR could possibly have influenced the performance of teams in Group A. To strengthen reliability, mannequin-based data of CPR performance should be used in addition to observational data [39]. The video observations in this study demonstrate that the raters assessed some aspects of D-CPR performance in different ways, indicated by some large inter-observer differences. The use of medical devices (e.g. bag-mask, oro-pharyngeal airway and backboard) is easy to assess, whereas aspects of D-CPR such as "checked response by shaking" and "checked breathing for a max. 10 s." depends on the individual investigators' judgment and interpretation. The difference in two time variables (item 20 and 21) between the two raters probably means that defining the exact point of time for discovering unconsciousness is different. These results are contrary to the findings in a study that assessed Advanced Life Support competence and found good reliability of the scores [40]. One reasonable explanation of the variations in inter-rater agreement in this study is that the two raters did not make a consensus scoring after the first individual assessment as in Ringsted et al. [40].

## Conclusion

This study revealed that observing training of other teams and participating in the debriefing did not itself improve performance of D-CPR in nursing student teams. The findings call for more time for repetitive practice and reflection and highlight that the most important aspects of D-CPR, like early defibrillation and hands-off time in relation to shock, must be emphasised during team training of nursing students.

## Endnote

^a^http://faculty.vassar.edu/lowry/VassarStats.html accessed in January 2012.

## Competing interests

SEH is employed part-time (25%) as Training Coordinator of University Programs at Stavanger Acute Medicine Foundation for Education and Research (SAFER). CAB is employed part-time (20%) as a facilitator at SAFER. ES is medical director at SAFER. SEH has received financial support from the Laerdal Foundation for Acute Medicine but otherwise the authors declare they have no competing interests and no financial disclosures.

## Authors' contributions

SEH participated in the design and planning of the study and the development of the D-CPR checklist; collected the data, assessed the video recordings, performed the descriptive statistics, wrote the manuscript draft, and coordinated the subsequent versions of the manuscript. CAB participated in the development of the D-CPR checklist, assessed the video recordings, performed the statistical analysis, and made important additions in drafting the manuscript. HR and FF participated in the design and planning of the study, revised the study manuscript and made important additions. ES participated in the design and planning of the study and was involved in drafting and revising the manuscript. All authors have reviewed the submitted version. All authors read and approved the final manuscript.

## Supplementary Material

Additional file 1 **The D-CPR checklist**. Click here for file

Additional file 2 The number of nursing student teams (n = 28) that followed the D-CPR checklist, number of items with agreement and disagreement between the two raters, mean (*median) values of time variables and kappa with linear weighting. Click here for file

Additional file 3 **Differences between rater 1 and rater 2 in time variables, item 20 Time (sec.) from discovery of unconsciousness until chest compressions started, 21 Time (sec.) from discovery of unconsciousness until shock was delivered and 24 Hands-off time (sec.) in relation to first shock**. Click here for file
