# Supplementary material for: A comparative study of defibrillation and cardiopulmonary resuscitation performance during simulated cardiac arrest in nursing student teams
Source: Scand J Trauma Resusc Emerg Med. 2012 Apr 2;20:23. doi: 10.1186/1757-7241-20-23 (PMC3361478; doi:10.1186/1757-7241-20-23)
Supplement: Additional file 1 — The D-CPR checklist. [file 1757-7241-20-23-S1.DOCX]

**Appendix 1**

The D-CPR checklist

| **No** | **Items** |  |
| --- | --- | --- |
| 1 | Checked response verbally | Yes/No |
| 2 | Checked response by shaking | Yes/No |
|  | **Examination** |  |
| 3 | Opened the airways | Yes/No |
| 4. | Checked breathing for a max. 10 sec. | Yes/No |
| 5 | Verbally stated cardiac arrest | Yes/No |
| 6 | Did not check pulse | Yes/No |
| 7 | Called 113 | Yes/No |
|  | **Performance of chest compressions** |  |
| 8 | Lowered the bed | Yes/No |
| 9 | Counted aloud | Yes/No |
| 10 | Stood on their toes | Yes/No |
| 11 | Kneeled on the bed | Yes/No |
| 12 | Applied the backboard | Yes/No |
|  | **Performance of ventilations** |  |
| 13 | Inserted an oro-pharyngeal airway | Yes/No |
| 14 | Placed the bag-mask | Yes/No |
| 15 | Applied 30:2 | Yes/No |
|  | **Use of semi-automatic defibrillator** |  |
| 16 | Put the semi-automatic defibrillator on the bed table | Yes/No |
| 17 | Attached pads | Yes/No |
| 18 | Said “all away from the bed/patient” | Yes/No |
| 19 | Performed “quick look” (that everybody was away) | Yes/No |
|  | **Time items** |  |
| 20 | Time from discovery of unconsciousness until chest compressions started | Time (seconds) |
| 21 | Time from discovery of unconsciousness until shock was delivered | Time (seconds) |
| 22 | Hands-off time from delivery of first shock until first compression was performed | Time (seconds) |
| 23 | Hands-off time from last compression until delivery of shock | Time (seconds) |
| 24 | Hands-off time in relation to first shock | Time (seconds) |
