# Supplementary material for: A comparative study of defibrillation and cardiopulmonary resuscitation performance during simulated cardiac arrest in nursing student teams
Source: Scand J Trauma Resusc Emerg Med. 2012 Apr 2;20:23. doi: 10.1186/1757-7241-20-23 (PMC3361478; doi:10.1186/1757-7241-20-23)
Supplement: Additional file 2 — The number of nursing student teams (n = 28) that followed the D-CPR checklist, number of items with agreement and disagreement between the two raters, mean (*median) values of time variables and kappa with linear weighting. [file 1757-7241-20-23-S2.DOC]

**Appendix 2.**

**The number of nursing student teams (n=28) that followed the D-CPR checklist, number of items with agreement and disagreement between the two raters, mean (*median) values of time variables and kappa with linear weighting.**

| No. | Items | Yes | No | Agree-ment | Disagree-ment | Time  (seconds) | *K* |
| --- | --- | --- | --- | --- | --- | --- | --- |
| 1 | Checked response verbally | 25 (89%) | 3(11%) | 28(100%) | 0 |  | 1.00 |
| 2 | Checked response by shaking | 12 (43%) | 7(25%) | 19(68%) | 9(32%) |  | 0.38 |
| 3 | Opened the airways | 4 (14%) | 21(75%) | 25(89%) | 3(11%) |  | 0.67 |
| 4 | Checked breathing for a max. 10 sec. | 7 (25%) | 10 (36%) | 17(61%) | 11 (39%) |  | 0.28 |
| 5 | Verbally stated cardiac arrest | 10 (36%) | 17 (61%) | 27(53%) | 1(3.5%) |  | 0.92 |
| 6 | Did not check pulse | 21 (75%) | 4 (14%) | 25(89%) | 3(11%) |  | 0.66 |
| 7 | Called 113 | 23 (82%) | 0 | 23(82%) | 5(18%) |  | n.c. |
| 8 | Lowered the bed | 4 (14%) | 21(75%) | 25(89%) | 3(11%) |  | 0.66 |
| 9 | Counted aloud | 26 (93%) | 1 (3.5%) | 27(96.5%) | 1(3.5 %) |  | 0.65 |
| 10 | Stood on their toes | 3 (11%) | 15 (53%) | 18(64%) | 10 (36%) |  | 0.24 |
| 11 | Kneeled on the bed | 15 (53%) | 11 (39%) | 26(92%) | 2(8%) |  | 0.85 |
| 12 | Applied the backboard | 27(96.5%) | 0 | 27(96.5%) | 1(3.5%) |  | 0.00 |
| 13 | Inserted an oro-pharyngeal airway | 13 (46%) | 14 (50%) | 27(96%) | 1 (4%) |  | 0.93 |
| 14 | Placed the bag-mask | 27(96.5%) | 0 | 27(96%) | 1(3.5%) |  | 0.00 |
| 15 | Applied 30:2 | 28 (100%) | 0 | 28(100%) | 0 |  | n.c. |
| 16 | Put the semi-automatic defibrillator on the bed table | 17 (61%) | 11(39%) | 28(100%) | 0 |  | 1.00 |
| 17 | Attached pads | 25 (89%) | 0 | 25(89%) | 3 (11%) |  | n.c. |
| 18 | Said “all away from the bed/patient” | 11 (39%) | 10 (36%) | 21(75%) | 7 (25%) |  | 0.51 |
| 19 | Performed “quick look” (that everybody was away) | 20 (72%) | 4 (14%) | 24(86%) | 4 (14%) |  | 0.58 |
|  | Total numbers of assessment | 318 (59 %) | 149  (28.0%) | 467  (88%) | 65 (13%) |  |  |
|  | **Time items** |  |  |  |  |  |  |
| 20 | Time from discovery of unconsciousness until chest compressions started |  |  |  |  | 37 |  |
| 21 | Time from discovery of unconsciousness until shock was delivered |  |  |  |  | 147 |  |
| 24 | Hands-off time in relation to first shock |  |  |  |  | *33 |  |

n.c.= not calculated. This quantity cannot be calculated
