# Supplementary material for: A comparative study of defibrillation and cardiopulmonary resuscitation performance during simulated cardiac arrest in nursing student teams
Source: Scand J Trauma Resusc Emerg Med. 2012 Apr 2;20:23. doi: 10.1186/1757-7241-20-23 (PMC3361478; doi:10.1186/1757-7241-20-23)

**Appendix 3.**

**Differences between rater 1 and rater 2 in time variables, item 20 *Time (sec.) from discovery of unconsciousness until chest compressions started*, 21 *Time (sec.) from discovery of unconsciousness until shock was delivered* and 24 *Hands-off time (sec.) in relation to first shock***


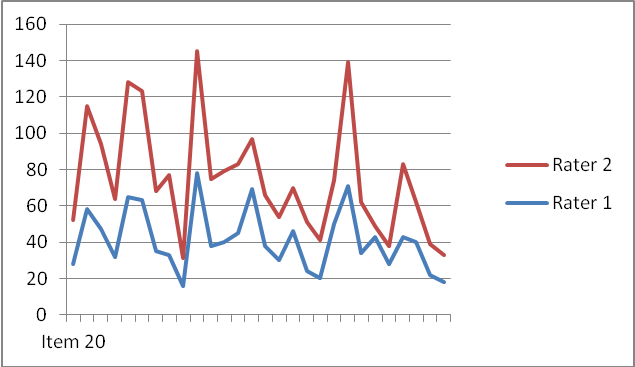


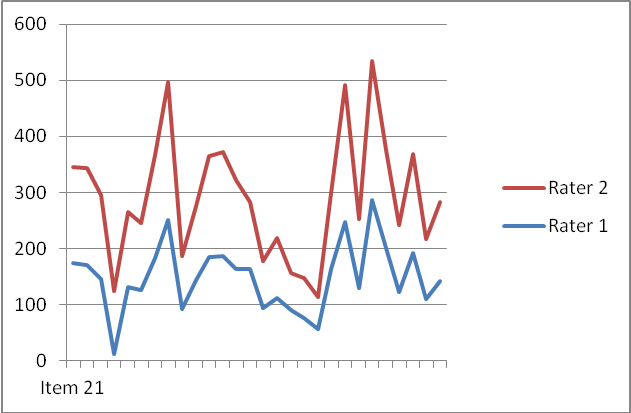


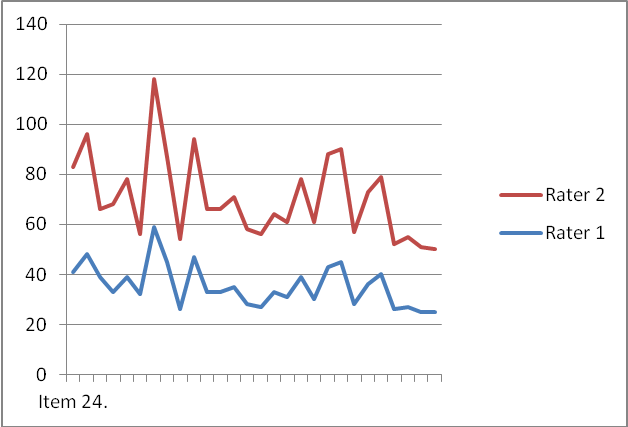

Supplement: Additional file 3 — Differences between rater 1 and rater 2 in time variables, item 20 Time (sec.) from discovery of unconsciousness until chest compressions started, 21 Time (sec.) from discovery of unconsciousness until shock was delivered and 24 Hands-off time (sec.) in relation to first shock. [file 1757-7241-20-23-S3.DOC]
